# Recent Advances in Polymer-Based Thermal Barrier Materials for Mitigating Thermal Runaway Propagation in Lithium-Ion Batteries

**DOI:** 10.3390/polym18070801

**Published:** 2026-03-26

**Authors:** Yang Li, Yong-Yan Xie, Yu-Jie Zhang, Lin Ma, Dun-Peng Bao, Su-Hang Wen, Shuai-Chi Liu, Zuan-Yu Chen, Guo-Dong Zhang, Xiao-Bo Ji, Long-Cheng Tang

**Affiliations:** 1 College of Chemistry and Chemical Engineering, Central South University, Changsha 410083, China; 2 Key Laboratory of Organosilicon Chemistry and Material Technology, Ministry of Education, Zhejiang Key Laboratory of Organosilicon Material Technology, College of Material, Chemistry and Chemical Engineering, Hangzhou Normal University, Hangzhou 311121, China

**Keywords:** polymer, thermal barrier, thermal runaway propagation, lithium-ion battery, battery safety

## Abstract

Thermal barrier materials play a crucial role in reducing heat transfer, suppressing thermal runaway (TR) propagation, and mitigating the risk of fire and explosion. Among the various types of thermal barrier materials, polymer-based thermal barrier materials, including polyimide (PI), aramid, epoxy resin (ER), polyurethane (PU), phenolic resin (PR), and silicone, have been widely applied in lithium-ion battery (LIB) safety protection owing to their excellent thermal stability, structural tunability, and favorable processability. This review provides a systematic and comprehensive overview of polymer-based thermal barrier materials for mitigating thermal runaway propagation in LIBs. The propagation pathways of TR in battery systems are first outlined to clarify the functional requirements of thermal barrier materials. Subsequently, representative classes of polymer materials are reviewed with emphasis on their structural characteristics and advantages. Strategies for enhancing thermal insulation, flame retardancy, heat absorption capacity, and mechanical robustness are then summarized in the context of thermal safety protection. Finally, key challenges associated with polymer-based thermal barrier materials are discussed, and future development directions are proposed.

## 1. Introduction

**Figure 1:**
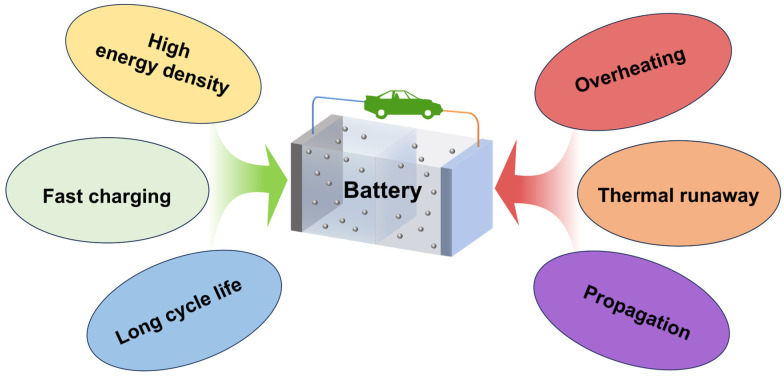
Schematic illustration of performance and safety trade-off in LIBs: enhanced energy density, fast-charging capability, and cycle life are associated with increased risks of overheating, thermal runaway, and propagation.

With the growing demand for extended driving range in electric vehicles, lithium-ion batteries (LIBs) are being developed toward higher volumetric energy densities [1,2,3]. However, intensified heat generation and accumulation during operation pose a higher risk of battery overheating and thermal runaway (TR) [4,5,6]. Moreover, owing to the intrinsically flammable nature of key battery components, such as electrolytes, separators, and electrodes, LIBs are prone to thermal runaway propagation once TR is initiated (Figure 1) [7,8,9,10]. In the absence of effective protective measures, such propagation can lead to fires and explosions, posing serious threats to the safety of human life and property. Therefore, developing effective strategies to suppress TR propagation is crucial for reducing the thermal hazards of LIBs in practical applications. In addition, next-generation battery systems such as semi-solid-state and solid-state batteries are expected to improve intrinsic safety by reducing or replacing the use of flammable liquid electrolytes with quasi-solid or solid electrolytes [11,12]. However, next-generation batteries still face several challenges, including lithium dendrite growth, interfacial instability, and localized heat accumulation, which may trigger internal short circuits and thermal runaway [13]. To address these risks, internal safety approaches based on thermal switch polymers have attracted increasing attention [14,15]. These materials undergo phase transitions at critical temperatures, sharply decreasing ionic conductivity to interrupt electrochemical reactions [16]. Nevertheless, while internal measures are vital, external thermal barrier materials remain essential for suppressing thermal propagation between cells and ensuring the overall safety of advanced battery modules.

So far, thermal barrier materials have emerged as a promising solution for suppressing TR propagation by effectively interrupting heat transfer pathways in LIBs [17,18,19]. Traditional thermal barrier materials, such as silica aerogels, ceramic fiber felts, and other inorganic insulation layers, exhibit excellent thermal insulation performance and high-temperature stability [20,21,22]. However, their practical application in LIBs is often constrained by inherent drawbacks. For example, silica aerogels possess highly porous nanostructured networks with abundant structural defects and poor continuity, which makes them particularly vulnerable to irreversible structural damage or even collapse under external mechanical stress, leading to a significant degradation of their thermal insulation performance [23,24,25]. Ceramic fiber mats are typically composed of loosely entangled fibers with limited inter-fiber bonding, resulting in fiber fracture, delamination, and structural densification under mechanical compression or vibration, ultimately leading to reduced thermal insulation reliability [26,27,28]. Similarly, conventional inorganic insulation layers often exhibit intrinsic brittleness and weak interfacial adhesion, which compromise their structural integrity and long-term durability under thermo-mechanical conditions [29]. In contrast, polymer-based thermal barrier materials offer distinct advantages, such as structural tunability and favorable processability, which enable rational material design and multifunctional integration for performance optimization [30]. As a result, these features make them a promising material platform for mitigating TR propagation and enhancing the overall safety of LIB systems.

Indeed, a wide range of polymer-based thermal barrier materials have been reported in recent years [31,32,33,34,35]. However, based on a survey of recent reviews on polymer-based thermal barrier materials, there has not been a comprehensive and critical review focusing on advances in polymer-based thermal barrier materials for mitigating TR propagation in LIBs over the past decade, particularly regarding emerging materials, quantitative performance comparison, and updated mechanistic understanding. In this regard, we present a comprehensive and timely overview of polymer-based thermal barrier materials for LIB safety. Firstly, the fundamental propagation pathways of TR in battery systems are summarized, providing a basis for understanding the design requirements of thermal barrier materials, and the corresponding design principles for suppressing TR propagation are established. Next, representative polymer-based thermal barrier materials are systematically reviewed, with a focus on typical examples from thermoplastic and thermosetting polymers, highlighting their material compositions, structural characteristics, and multifunctional performance in suppressing TR propagation. Moreover, functional optimization strategies for polymer-based thermal barrier materials are discussed, including thermal insulation, heat absorption, flame retardancy, and mechanical robustness, with representative examples and comparative analysis. Finally, we provide perspectives regarding the challenges and opportunities of developing high-performance polymer-based thermal barrier materials. We anticipate that this review will provide useful insights for future developments in battery thermal protection and promote the practical application of high-safety LIB systems.

## 2. TR Propagation in LIBs

Understanding TR propagation in LIBs is essential for the rational design of effective thermal barrier materials. TR can be triggered under abnormal operating conditions, including mechanical, electrical, and thermal abuse [36,37,38]. Once triggered, unmitigated TR can rapidly propagate to adjacent cells, potentially giving rise to severe fire and explosion risks [39]. Therefore, clarifying TR propagation pathways provides the basis for establishing design principles of polymer-based thermal barrier materials for improved LIB safety.

### 2.1. Propagation Pathways

According to heat and mass transfer mechanisms, thermal runaway propagation in LIBs can be categorized into five pathways: heat conduction, heat convection, heat radiation, jet fire, and vented gases [40,41]. When a single cell undergoes thermal runaway, rapid gas generation elevates the internal pressure, which can trigger vent rupture [42]. Under severe conditions, the cell casing may further rupture, ejecting combustible gases, electrolyte vapour, and particulate matter at high velocity to form a venting jet [43]. After mixing with ambient air to reach the flammability limits, the jet can be ignited by the hot cell surface or other external ignition sources, thereby producing a jet fire [44]. In a battery pack composed of densely arranged cells, the heat released from a failed cell can be transferred to neighbouring cells via conduction, convection, and radiation [45]. Meanwhile, the high-velocity jet and entrained particles intensify local convective heat transfer and generate thermal shock, thereby promoting cell-to-cell thermal coupling and fault propagation [46].

### 2.2. Design Principles for Polymer-Based Thermal Barrier Materials

**Figure 2:**
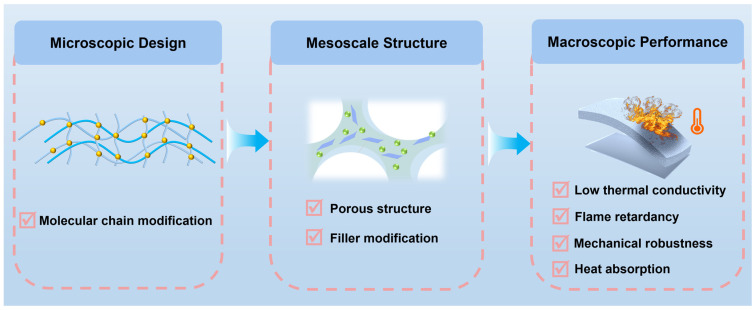
Schematic illustration of the multiscale design of polymer-based thermal barrier materials for LIB safety.

Based on the propagation pathways and key characteristics of TR discussed above, polymer-based thermal barrier materials should satisfy several fundamental requirements to reliably mitigate TR propagation in LIBs. Effective thermal insulation is essential because conduction and convection are primary routes for heat transfer between neighbouring cells [47]. Thus, the barrier layer should exhibit low thermal conductivity and maintain stable insulating performance at elevated temperatures. Moreover, radiative heating and flame impact can rapidly increase the temperature of adjacent cells [48]. Therefore, the barrier material should limit radiative heat absorption while retaining thermal and structural stability under high heat flux. Additionally, vented gases and jet flames introduce flammable species and toxic smoke, posing severe fire hazards [49]. Accordingly, the insulation layer should exhibit effective flame retardancy. They also require sufficient mechanical robustness because TR propagation is often accompanied by thermal shock, which can cause cracking or structural collapse [50]. Overall, these requirements clarify the key properties for polymer-based thermal barrier materials and guide the discussion of representative material systems in the following sections. To further illustrate the structure–property relationship between multiscale structural design and macroscopic performance, a schematic framework is presented in Figure 2.

## 3. Advanced Polymers for Thermal Barrier in LIBs

For external thermal barrier applications in LIBs, polymers must possess intrinsic high-temperature stability, low heat transfer capability, and robust structural integrity under extreme thermal conditions. At present, polyimides (PIs), aramids, epoxy resins (ER), polyurethanes (PU), phenolic resins (PR), and silicones are commonly used as thermal barrier materials in LIBs [51,52,53,54,55,56]. The thermal conductivity, flame retardant rating, and mechanical strength of the selected representative polymer-based thermal barrier materials used in LIBs are summarized in Table 1. It should be noted that discrepancies among literature data may arise from differences in intrinsic material properties, structural characteristics, material thickness, testing configurations, and inconsistent definitions of TR related metrics. Therefore, direct comparison of reported results should be made with caution.

### 3.1. PI

PIs are typically polymerized in polar solvents using aromatic dianhydrides and diamines as monomers through polycondensation [59]. Since Bogert et al. first reported polyimides in 1908, they have attracted considerable interest from both academia and industry [60]. At present, polyimide synthesis is generally categorized into two approaches: one-step imidization and the two-step poly (amic acid) route [61]. In the one-step route, the monomers are polymerized in high-boiling aprotic polar solvents (e.g., N-methyl-2-pyrrolidone or dimethyl sulfoxide) under elevated temperatures, where poly (amic acid) segments formed transiently are converted to the imide structure via in situ imidization [62]. Notably, triethylamine and related catalysts are frequently used to promote the reaction kinetics and improve efficiency [63]. Although the one-step process is procedurally simple, precise control over monomer reactivity is required. Otherwise, a broad molecular-weight distribution may arise, leading to less uniform performance. By contrast, the two-step route is more widely adopted because it offers superior controllability over product quality [64]. In this method, poly (amic acid) is initially obtained under low-temperature conditions, followed by stepwise heating to promote cyclodehydration and generate the imide structure. Compared with the one-step route, the two-step process generally achieves higher imidization with fewer structural defects, thereby improving property uniformity and overall performance.

Owing to their inherent molecular architecture, polyimides feature an intrinsically rigid backbone and strong intermolecular interactions, thereby endowing the material with outstanding properties [65]. Specifically, the alternating arrangement of rigid aromatic rings and relatively flexible ether bonds forms a distinctive segment structure, thereby providing excellent thermal stability. In addition, the conjugated backbone composed of phenyl and imide rings creates a highly rigid molecular framework, which substantially suppresses the movement of chain segments and consequently enhances mechanical robustness. As a result, polyimide has been widely considered a promising polymer for thermal-barrier protection in LIBs. For example, Li et al. synthesized a porous PI aerogel interlayer via freeze-drying of a poly(amic acid) salt precursor and subsequent stepwise thermal imidization [51]. The aerogel with a thickness of 2 mm extended the TR propagation time between adjacent cells from 10 s to 595 s, maintained non-ignition for 900 s, and significantly reduced heat release. Hou et al. prepared PI nanofiber-reinforced PI aerogel membranes through sol–gel gelation followed by supercritical carbon dioxide drying [66]. The resulting membranes exhibited an ultralow thermal conductivity of 0.0279 W m^−1^ K^−1^ while maintaining excellent mechanical robustness, with a tensile strength of 2.95 MPa and 86.1% strength retention after 1000 folding and unfolding cycles. With efficient thermal insulation and foldable durability, these PI aerogel membranes could serve as flexible thermal barrier interlayers to improve LIBs safety. Zhang et al. synthesized flexible PI foams via a green aqueous hydrogel strategy that builds ultra-strong hydrogen-bonding network backbones [67]. The resulting PI foams exhibited low thermal conductivity (~0.048 W m^−1^ K^−1^) and a high compressive modulus (11.17 MPa), while still maintaining 4.21 MPa in compressive modulus (≈91% retention) after heat treatment at 300 °C. Accordingly, these PI foams may serve as lightweight protective insulation layers for external battery packaging.

### 3.2. Aramid

Aramids, a class of polymers containing multiple aromatic amide bonds connecting aromatic rings, were developed in the 20th century [68]. Conventional routes to aramids primarily include low-temperature solution polycondensation, interfacial polycondensation, and vapor-phase polymerization [69,70,71]. Low-temperature solution polycondensation for aramid synthesis is typically performed in highly polar aprotic solvents (e.g., N-methyl-2-pyrrolidone, N,N-dimethylacetamide, or hexamethylphosphoramide), where p-phenylenediamine and terephthaloyl chloride undergo step-growth polycondensation in a low-temperature batch condition [72]. Nevertheless, interfacial polycondensation is often limited due to the highly exothermic reaction and the environmental and safety concerns posed by toxic solvents. Interfacial polycondensation offers rapid reaction kinetics and straightforward phase separation. However, limitations in continuous processing have restricted large-scale industrialization. Vapor-phase polymerization offers extremely fast kinetics under inert atmospheres, while safety risks associated with volatile acid chlorides at elevated temperatures also pose significant challenges for industrial scale-up. To address these limitations, a series of emerging synthetic strategies have been explored in recent years, including n-pentane-assisted polycondensation, nonaqueous suspension polycondensation, ionic-liquid-mediated routes, and microchannel reactor-based synthesis, aiming to improve process safety, controllability, and sustainability [73,74,75,76].

Aramids are generally classified into two major types according to their backbone structures: para-aramids and meta-aramids [77]. In para-aramids, amide linkages alternate with phenylene units along the main chain, resulting in a highly linear and rigid architecture [78]. The amide–aromatic conjugation enhances backbone planarity and suppresses internal rotation, thereby reducing free volume and limiting segmental mobility, which translates into high intrinsic strength. Moreover, strong interchain hydrogen bonding builds a robust physical network and promotes orderly chain packing and orientation, further increasing crystallinity and molecular alignment and ultimately delivering a high modulus and excellent mechanical performance [79]. Compared with para-aramids, meta-aramids feature a non-linear, “zigzag” backbone arising from meta-substitution, which increases conformational freedom and thus enhances chain flexibility [80]. Meanwhile, strong intermolecular interactions associated with the aromatic backbone and amide groups effectively suppress segmental thermal motion, leading to a relatively high glass-transition temperature. Owing to these structural attributes, meta-aramids generally exhibit excellent thermal stability, inherent flame retardancy, and favorable electrical insulation, making them well suited for high-temperature protection and related applications [81]. For example, Wu et al. reported a lightweight aramid-derived structural composite constructed by infiltrating a shear-stiffening gel into an aramid nanofiber aerogel scaffold followed by orthogonal lamination [52]. Benefiting from the retained microvoids within the densified aramid nanofiber network, the composite achieved an ultralow thermal conductivity of 0.09 W m^−1^ K^−1^ and maintained effective thermal insulation over a wide temperature window, ranging from approximately −120 to 300 °C. Meanwhile, the gel-assisted laminated architecture enabled efficient impact-energy dissipation through synergistic mechanisms such as structure hardening, interlayer sliding, and intralayer cracking, highlighting the suitability of aramid-based architectures for coupled thermal–mechanical protection.

### 3.3. ER

Epoxy resins are a class of thermosetting resins characterized by the presence of two or more epoxy groups, which can be cured to form three-dimensional crosslinked networks [82]. Its research and application can be traced back to the late 19th century, when the German chemist Lindmann obtained a viscous product from the reaction between hydroquinone and epichlorohydrin [83]. Subsequent observations indicated that the resinous product was curable with acid anhydrides, yielding a hardened material [84]. As curing mechanisms became better established, epoxy resins were shown to crosslink efficiently in the presence of curing agents such as organic amines and dibasic acids, resulting in cured networks with high adhesive strength and good performance [85]. These findings laid the foundation for epoxy resins to emerge as a mainstream engineering thermoset, enabling widespread research and commercialization.

The unique molecular structure and cross-linking network endow epoxy resins with a series of excellent physicochemical properties, including outstanding mechanical strength, excellent chemical resistance, strong adhesion, low curing shrinkage, and good electrical insulation [86]. Therefore, epoxy resins are well-suited for LIB systems, which require durable mechanical integrity and strong interfacial bonding under typical operating conditions such as vibration, shock, and thermal cycling. For example, Wang et al. fabricated epoxy-resin barrier plates by incorporating a surface-modified MXene flame-retardant additive into epoxy and casting plates for NCM523 pouch cells [87]. In TR propagation tests, the barrier extended the time interval between the TR events of two adjacent cells from 202 s to 272 s, indicating a more effective delay of TR propagation. Huang et al. developed a flame-retardant epoxy resin by covalently integrating trithiocyanuric acid into the crosslinked network and then fabricated epoxy-based phase-change barrier sheets with improved flame resistance and stability [53]. The modified epoxy skeleton reduced the peak heat release rate to below 300 kW m^−2^ and markedly suppressed smoke release. Thermal-runaway propagation was completely prevented by the 3 mm-thick barrier in a multi-cell module, and the protected cell reached a peak temperature of only 105 °C under abuse conditions. Wang et al. developed a fire-resistant epoxy barrier by incorporating phosphorus-doped MoS_2_ nanorods obtained through hydrothermal conversion and subsequent high-temperature calcination [88]. When a 3 mm-thick barrier was applied, thermal runaway was not triggered in the adjacent cell, and the surviving cell retained largely intact internal structure and electrochemical characteristics, indicating effective suppression of TR propagation.

Benefiting from a highly crosslinked structure, epoxy resins typically exhibit relatively high glass transition temperatures and can maintain dimensional stability until the onset of network degradation at elevated temperatures [89]. However, epoxy resins are intrinsically flammable and can exhibit high heat release once ignited, which limits their direct use as thermal barriers for suppressing TR propagation. Despite their moderate thermal insulation capability, further functional modification is still necessary to achieve practical safety performance. Therefore, epoxy resins used for battery protection often require the synergistic integration of flame retardant and thermally insulating designs, as they provide complementary functionalities in reducing thermal feedback, delaying ignition, and maintaining structural integrity.

### 3.4. PU

PU is a widely used multifunctional polymer synthesized via step-growth polymerization through reactions between diisocyanates (or polyisocyanates) and diols (or polyols), forming polymer chains containing urethane groups [90]. The molecular structure of polyurethane consists of alternating hard and soft segments, each of which contributes to its thermal properties. Specifically, hard segments with higher glass transition temperatures primarily determine the stiffness and mechanical integrity of polymers, while soft segments with lower glass transition temperatures modulate their elastic behavior [91]. Owing to their segmented molecular structure, polyurethanes simultaneously achieve wear resistance, mechanical toughness, low-temperature flexibility, chemical resistance, and processability [92]. These intrinsic properties endow polyurethanes with exceptional versatility, making them highly attractive polymeric materials for a broad range of applications. Recent efforts have repurposed polyurethane into thermal barrier materials for high-value engineering applications. For instance, Yang et al. reported flame-retardant polyurethane composites designed as protective layers for battery modules [93]. When integrated into battery assemblies, these polyurethane-based barriers effectively suppressed heat release and smoke generation during thermal abuse, thereby significantly prolonging the thermal runaway propagation barrier time. This work demonstrates the feasibility of using polyurethane composites as external thermal barrier components to mitigate thermal runaway propagation at the module level. Han et al. developed an intrinsically flame-retardant polyurethane-based solid–solid phase change material, in which flame-retardant functionalities were incorporated into the polymer backbone through chemical modification [94]. The resulting material combined latent heat storage with fire resistance, enabling both temperature regulation during battery operation and effective suppression of thermal runaway, as evidenced by delayed ignition and reduced peak temperatures in battery abuse tests. Luo et al. designed cross-linked polyurethane phase change materials specifically for battery jacket and encapsulation applications [54]. These polyurethane-based materials exhibited stable thermal regulation capability under high-rate cycling conditions while simultaneously enhancing fire resistance, indicating their potential as multifunctional thermal barrier layers for external battery protection.

### 3.5. PR

PRs are typically synthesized via polycondensation reactions between phenolic compounds and aldehydes at controlled molar ratios under catalytic conditions and elevated temperatures [95]. The molecular backbone of phenolic resins contains a high density of stacked aromatic rings, resulting in strong intermolecular cohesion and a highly crosslinked network structure. Consequently, phenolic resins exhibit excellent chemical resistance, high char yield, and good mechanical strength, which have enabled their widespread application in flame-retardant and thermal protection materials [96]. For instance, Zhang et al. reported phenolic resin-based aerogel materials exhibiting excellent thermal stability and flame resistance, which maintained structural integrity under severe fire and high-temperature conditions [97]. These phenolic aerogels leveraged the highly crosslinked aromatic network and high char yield of phenolic resins, demonstrating their suitability for advanced thermal protection and fire-resistant applications in harsh environments. Building on these intrinsic fire-protection advantages, phenolic resin systems have recently been extended to lithium-ion battery safety applications. Li et al. integrated phenolic resin-based aerogel components as thermal barrier layers within electric vehicle battery packs [55]. Under thermal runaway conditions, the phenolic resin aerogel was employed as a structural thermal barrier within the battery pack, acting as fire-resistant thermal barriers that physically isolated adjacent cells. Benefiting from its exceptional thermal stability, with structural integrity maintained even under flame exposure exceeding 1300 °C, the aerogel effectively delayed heat transfer and restrained fire spread at the pack level.

### 3.6. Silicone

Silicones consist of Si–O–Si backbones with pendant organic groups, which can adopt either linear chains or three-dimensionally crosslinked networks [98]. At present, silicone rubber is the most widely used class of silicone materials, in which the backbone silicon atoms can bear various organic groups, such as methyl, phenyl, or trifluoropropyl groups [99]. Among these, the most common commercial silicone rubber systems are typically methyl-substituted, with a small fraction of vinyl functionalities introduced to provide reactive sites for crosslinking. The Si–O bond energy (460 kJ mol^−1^) is significantly higher than that of typical C–C bonds (356 kJ mol^−1^) found in other polymer backbones [100]. In addition, the large electronegativity difference between silicon and oxygen results in a highly polar siloxane bond, which is commonly described as possessing partial ionic properties. These bonding features provide the basis for the thermal and oxidative stability of silicone rubbers [101]. Consequently, silicone rubbers generally retain molecular integrity during prolonged exposure at approximately 150 °C and exhibit acceptable structural stability during short-term exposure to higher temperatures [102]. In addition, silicone rubbers show resistance to oxygen and ozone, leading to reduced oxidative ageing [103]. Moreover, the flexibility of the siloxane backbone and weak intermolecular interactions are associated with a low glass-transition temperature, high elasticity, and good compression resilience over a wide temperature range [104]. These properties enable silicone rubber to maintain excellent performance under a variety of extreme environmental conditions.

In recent studies, silicone rubber has emerged as a promising thermal barrier material for suppressing thermal runaway propagation in LIBs. For example, Wang et al. reported a silicone rubber thermal barrier with temperature-responsive characteristics, in which the material dynamically adjusted its thermal regulation behaviour during battery overheating. This adaptive thermal buffering effect effectively delayed heat accumulation and prevented thermal runaway propagation between neighbouring cells under abuse conditions [105]. Chen et al. designed a ceramifiable silicone rubber foam that undergoes in situ ceramic transformation upon exposure to high temperatures, enabling the material to retain structural integrity and form a continuous thermal barrier under flame and abuse conditions. As a result, the silicone rubber foam effectively isolated heat transfer between adjacent cells and significantly inhibited thermal runaway propagation at the module level [56]. Cui et al. developed a multifunctional silicone rubber foam incorporating heat-absorption capability, which combined low thermal conductivity with pronounced thermal buffering during high heat flux exposure. Module-level tests demonstrated that a 4 mm-thick silicone rubber barrier completely suppressed cascading thermal runaway, outperforming conventional insulation materials that relied solely on thermal resistance [106]. These advancements in silicone rubber materials underscore their potential as effective thermal barriers for LIBs safety applications.

### 3.7. Others

While high-performance engineering polymers mentioned above offer tailored functional properties for battery protection, other polymers such as polypropylene (PP) and styrene–butadiene–styrene (SBS) are gaining increasing attention due to their excellent processability and cost-effectiveness. For instance, Chen et al. demonstrated that a 2 mm flame-retardant PP barrier can significantly prevent TR propagation in 18650 cell modules [57]. Yang et al. developed an excellent barrier using SBS as the supporting matrix combined with paraffin and triphenyl phosphate. The results showed that a 3 mm thick SBS-based barrier demonstrated effective heat suppression and mitigated TR propagation in a 26650 battery module under simulated abuse conditions [58].

**Figure 3:**
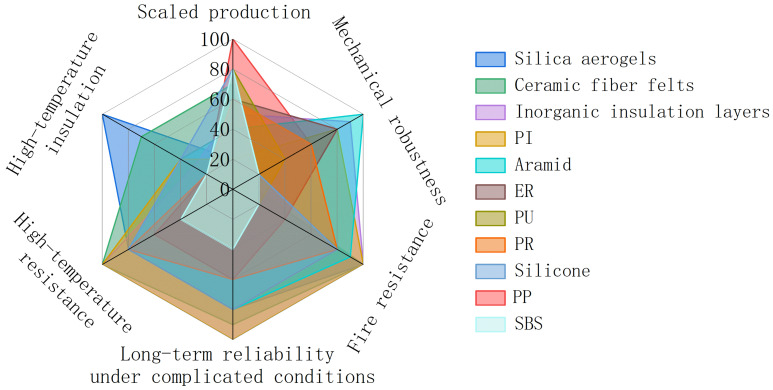
Radar chart comparing the performance of representative thermal barrier materials in terms of scalable production, mechanical robustness, fire resistance, long-term reliability, high-temperature resistance, and thermal insulation [23,27,29,57,58,65,81,86,92,96,104].

In order to provide a comprehensive evaluation, Figure 3 compares the polymers discussed above with silica aerogels, ceramic fiber felts, and inorganic insulation layers that represent current industrial applications for thermal protection [23,27,29,57,58,65,81,86,92,96,104]. The conventional inorganic materials, such as silica aerogels, ceramic fiber felts, and inorganic insulation layers, generally exhibit superior high-temperature resistance and thermal insulation performance, whereas their mechanical robustness and scalability are relatively limited. In contrast, polymers such as PI, aramid, ER, PR, and silicone demonstrate a more balanced combination of thermal stability and mechanical robustness. Other polymers like PU, PP and SBS offer distinct advantages in scalability. However, trade-offs among different properties remain inevitable, particularly between thermal insulation, flame retardancy, and mechanical robustness.

**Figure 4:**
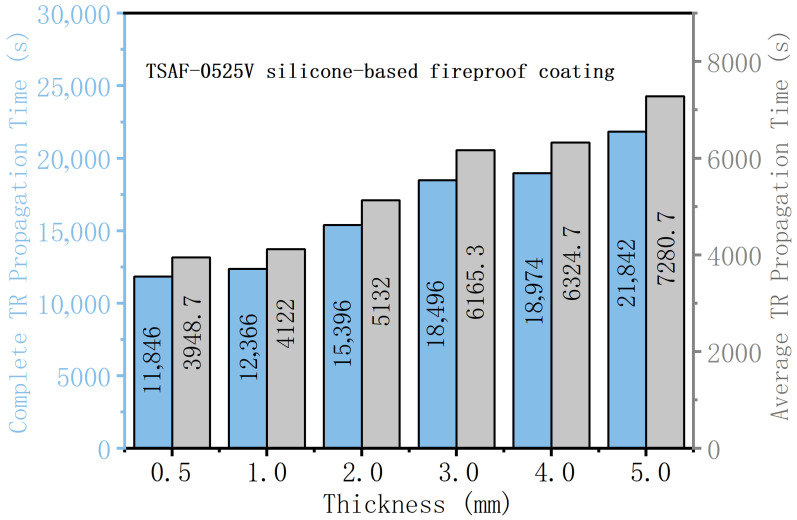
Relationship between the thickness of the TSAF-0525V silicone-based fireproof coating and TR propagation time, including the complete propagation time (from the maximum temperature of battery 1 to that of battery 4) and the average propagation time for modules with different coating thicknesses. Data adapted from [107].

The thickness of thermal barrier materials is also a critical parameter in suppressing TR propagation. Previous studies have demonstrated that increasing the thickness of the thermal barrier can significantly prolong TR propagation time [107]. As summarized in Figure 4, increasing the thickness of the TSAF-0525V silicone-based fireproof coating leads to longer complete propagation time and average propagation time, indicating enhanced suppression of TR propagation. However, excessive thickness may compromise weight, space utilization, and energy density, suggesting an inherent trade-off between safety and practical application in battery systems. This finding further emphasizes the importance of integrating material selection with structural design in developing effective thermal barrier systems.

## 4. Strategies to Enhance the Performance of Polymer-Based Thermal Barrier Materials

**Figure 5:**
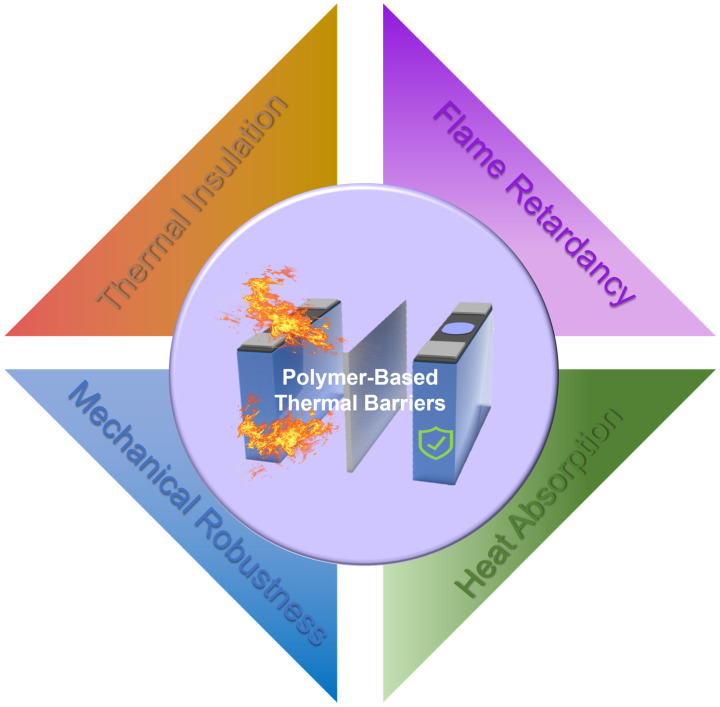
Design concept of polymer-based thermal barrier materials, including thermal insulation, heat absorption, flame retardancy, and mechanical robustness for battery safety protection.

As discussed above, TR events in LIB packs involve rapid temperature rise, high heat flux loading, and aggressive flame and jet impact. Such coupled thermo-mechanical conditions markedly raise the performance requirements for polymer-based thermal barrier materials. Therefore, polymer-based thermal barrier materials used for battery safety protection must meet more demanding performance requirements, including good thermal insulation capability, effective flame retardancy, and mechanical stability under elevated temperatures and impact conditions. In this section, we systematically summarize representative design strategies to enhance the performance of polymer-based thermal barrier materials from four aspects: thermal insulation, heat absorption, flame retardancy, and mechanical robustness (Figure 5). It should be noted that relying solely on thermal insulation may lead to heat accumulation and temperature gradients during normal operation. Therefore, achieving balanced thermal management requires the synergistic integration of thermal insulation, heat absorption, and heat dissipation.

### 4.1. Thermal Insulation Strategies

#### 4.1.1. Porous Structure Design

The thermal insulation performance of polymer-based thermal barrier materials essentially relies on their ability to suppress heat transfer [108]. In dense polymers, heat is mainly conducted through the continuous solid framework, thereby limiting the extent to which the effective thermal conductivity can be reduced due to uninterrupted conduction pathways. Constructing polymers into porous structures can markedly lower the effective thermal conductivity [109]. On the one hand, porosity decreases the solid phase volume fraction and disrupts continuous heat-transfer pathways, thereby weakening solid-state conduction. On the other hand, the introduced gas phase possesses much lower thermal conductivity, further reducing the overall heat transfer. Therefore, designing polymers as porous thermal barrier layers (such as foam materials and aerogel materials) has become a common strategy for suppressing thermal runaway propagation in batteries. For example, Wu et al. introduced phenyl groups into the Si–O–Si backbone to impose steric hindrance, thereby optimizing the crosslinking and foaming reaction match during chemical foaming, enabling a rapid room-temperature fabrication of an ultralight phenyl-containing silicone foam with tunable pore structure [98]. Consequently, the sample can reduce the hot stage temperature from 180 °C to 55 °C, demonstrating markedly enhanced thermal insulation. Zhan et al. designed a lightweight PI/gelatin composite aerogel by ice-templated freeze-drying followed by thermal imidization [110]. The resulting closed pore aerogel structure reduced the thermal conductivity from 0.039 to 0.032 W·m^−1^·K^−1^, thereby enhancing the thermal insulation performance.

#### 4.1.2. Integration of Low Thermal Conductivity Materials

Materials with intrinsically low thermal conductivity play a vital role in regulating thermal insulation performance. Their incorporation into polymer matrices suppresses heat transfer and lowers the effective thermal conductivity. Commonly used low thermal conductivity fillers include silica aerogel particles, hollow glass microspheres, and vermiculite nanosheets [111,112,113]. For example, Kucharek et al. incorporated silica aerogel particles into epoxy via a delayed wet-mixing strategy, where the resin viscosity was rationally tuned during curing to minimize pore infiltration and preserve the nanoporous structure of aerogel, resulting in an over 40% reduction in thermal conductivity relative to pure epoxy [111]. Jeong et al. immobilized hollow glass microspheres onto glass fibers through polydopamine-assisted interfacial anchoring and fabricated the composites by liquid molding, the resulting composite exhibited a 14% improvement in thermal insulation performance compared with the unmodified glass fiber-reinforced polymer [114]. Sethurajaperumal et al. prepared exfoliated vermiculite nanosheets and formulated a vermiculite/epoxy nanocomposite coating, where vermiculite served as an insulating nanofiller owing to its intrinsically low thermal conductivity [115].

#### 4.1.3. Radiative Heat Transfer Suppression

At elevated temperatures, radiative heat transfer becomes more significant and can even dominate the overall heat flux, making radiation management essential for high temperature polymer-based thermal barrier design [116,117]. A widely adopted strategy involves incorporating radiation-suppressing fillers that reflect and scatter infrared radiation and enhance mid-infrared absorption or emissivity, thereby reducing net radiative heat transfer through the barrier [118,119]. Typical radiation-suppressing fillers include inorganic particles (e.g., Al_2_O_3_, TiO_2_, ZrO_2_) and carbon-based materials (e.g., graphene, carbon nanotubes), which can effectively reduce radiative heat transfer under high temperature [120,121,122,123,124]. This strategy is typically implemented by introducing high refractive index nanoparticles that boost infrared reflection and scattering, along with lamellar nanosheets that create tortuous optical pathways and multiple reflections to hinder infrared transmission. In porous polymers and aerogels, coupling these fillers with hierarchical pore structure can further suppress both conduction and radiation, thereby improving thermal insulation at elevated temperatures [125].

### 4.2. Heat Absorption Strategies

Currently, heat generated in the battery can be dissipated through endothermic phase-change processes, which buffer the temperature rise and weaken the conditions required for self-accelerating side reactions [126]. On the one hand, this heat absorption can interrupt subsequent exothermic side reactions in an abused cell, thereby lowering the probability of thermal runaway initiation and limiting its intensity. On the other hand, upon thermal runaway initiation, an endothermic barrier layer can provide thermal buffering by absorbing heat, reduce the thermal load on adjacent cells, and delay cell-to-cell propagation, thereby confining the thermal runaway event. Heat absorption strategies are typically realized by integrating phase change materials (PCMs) with polymer matrices to provide thermal buffering. For example, Chen et al. prepared a thermally conductive composite PCM by physically adsorbing paraffin wax onto expanded graphite and ceramic fillers, when applied to LIB thermal management, the optimized materials reduced cell temperature by 7.8 °C at 2C compared with natural convection [127]. Wang et al. constructed graphite powder with paraffin composite PCMs [128]. The maximum battery surface temperature decreased from 56.95 °C to 43.5 °C, demonstrating progressively enhanced heat-buffering capability. Chen et al. developed hybrid carbon-filled PCMs using few-layer graphene and graphite nanoplatelets to improve heat absorption, and the optimized formulation maintained the module temperature difference within 5 °C [129].

### 4.3. Flame Retardancy Strategies

The flame retardancy of polymer-based thermal barrier materials is critical for suppressing thermal runaway propagation and reducing the risk of fires and explosions. At present, polymer flame retardancy is commonly improved via two main routes, including intrinsic flame-retardant design and additive-type flame-retardant systems [130,131].

#### 4.3.1. Intrinsic Flame-Retardant Design

Intrinsic flame retardancy is typically achieved by copolymerizing flame-retardant units into the polymer backbone or by introducing them as pendant side groups along the polymer chains [132]. Due to these units are covalently immobilized and distributed uniformly at the molecular level within the matrix, intrinsically flame-retardant polymers often exhibit higher flame-retardant efficiency than conventional additive-based formulations. For example, to overcome the limitations of the epoxy resin with additive-type flame retardants, Yang et al. molecularly engineered an intrinsically flame-retardant bio-based epoxy monomer (MEP) by coupling lignin-derived vanillin with 4,4′-diaminodiphenylmethane and a reactive phosphorus unit, followed by epoxidation, and then co-curing MEP with a commercial bisphenol A diglycidyl ether epoxy resin to build a covalently integrated phosphorus/nitrogen-containing network [133]. The resulting intrinsic flame-retardant epoxy achieved UL-94 V0 at a low phosphorus loading. Wang et al. synthesized a reactive poly(ethylene methylphosphonothioate) (PEMPT), and chemically incorporated it into the polyurethane chains by partially replacing the conventional polyether polyol during one-pot foaming. The PEMPT-incorporated foams demonstrated substantially enhanced flame retardancy. With 2.5 wt% PEMPT in the polyol component, the foam achieved self-extinguishment within 3 s. At a higher loading of 10 wt% PEMPT, the limiting oxygen index increased to 23.5%, while the peak heat release rate and total heat release decreased by 25.8% and 24.0%, respectively, relative to the control foam [134]. Huang et al. achieved intrinsic flame retardancy in silicone rubber by using a P/N-containing siloxane crosslinker (BPTES) to covalently embed flame-retardant units into the cured network [135]. Compared with pristine silicone rubber, with the addition of BPTES reduced the peak heat release rate and peak smoke production rate by 55.9% and 48.6%, respectively, and HNTs@ZIF provided further reductions in total heat release and total smoke production.

#### 4.3.2. Additive-Type Flame-Retardant Systems

Additive-type flame-retardant systems are generally achieved by physically incorporating flame retardants or flame-retardant fillers into the polymer matrix [136,137]. Owing to their simplicity, broad applicability, and ease of processing, such approaches are among the most widely adopted flame-retardant strategies. According to chemical composition, commonly used flame retardants can be broadly classified into inorganic and organic types [138,139]. Inorganic flame-retardant fillers are extensively employed in polymer materials because of their advantages, including low cost, good processability, low toxicity, and effective smoke suppression [140,141]. Among them, aluminum hydroxide and magnesium hydroxide are the most representative and industrially mature inorganic flame retardants [142]. In addition, carbon-based flame retardants, such as graphite, as well as emerging two-dimensional materials, including black phosphorus and MXene, have attracted increasing attention owing to their unique structural features and multifunctional flame-retardant characteristics [143,144,145,146]. Organic flame retardants encompass a wide variety of compounds, and their development has largely evolved from halogen-based systems toward halogen-free flame-retardant strategies, particularly those based on phosphorus chemistry [147]. Although halogen-based flame retardants exhibit high flame-retardant efficiency, growing concerns over environmental pollution and toxicity have led to increasingly strict global regulations. Since the early 21st century, halogen-free flame retardants have become a research priority. Among them, organophosphorus flame retardants, represented by phosphine oxides, phosphonates, and phosphorus-containing heterocyclic compounds, can work synergistically in the gas phase and condensed phase, thereby providing excellent flame retardancy [148]. As a result, they have been widely applied in almost all classes of polymeric materials.

### 4.4. Mechanical Robustness

Mechanical robustness represents a prerequisite for polymer-based thermal barrier materials, as inadequate mechanical integrity fundamentally limits their practical applicability. The enhancement of polymer mechanical strength generally requires multiscale regulation from molecular interactions to network structures [149]. Introducing reversible intermolecular interactions, such as hydrogen bonding and metal coordination, strengthens interchain cohesion and enhances energy dissipation [150]. Meanwhile, tailoring the crosslinked network to be dynamically reconfigurable enables stress redistribution under mechanical loading, thereby potentially improving resistance to unpredictable mechanical failure [151]. Furthermore, composite modification, for example, through the incorporation of nanofillers, can promote load transfer and suppress crack propagation, leading to a synergistic enhancement of strength, toughness, and structural stability [152,153,154].

## 5. Conclusions and Perspective

In this review, we have summarized the fundamental propagation pathways of TR in battery systems and systematically discussed representative polymer-based thermal barrier materials, including polyimide, aramid, epoxy, polyurethane, phenolic resins, and silicone. Furthermore, strategies for enhancing thermal insulation, flame retardancy, heat absorption, and mechanical robustness have been comprehensively reviewed, providing guidance for the rational design of polymer-based thermal barriers for battery safety applications.

Although significant advancements have been made in advancing polymer-based thermal barrier materials for mitigating TR propagation in LIBs in recent years, substantial improvements are still required for practical application. Several critical challenges remain to be addressed in future research:

(1) Synergistic and balanced performance: Current polymer-based thermal barrier materials often exhibit inherent trade-offs among thermal insulation, flame retardancy, heat absorption, and mechanical properties. For instance, high loadings of flame retardants may deteriorate mechanical integrity, while the incorporation of heat absorbing components can compromise structural stability. Therefore, achieving a synergistic enhancement in flame retardancy, mechanical robustness, and long-term durability without sacrificing low thermal conductivity remains a critical direction for future research.
(2) Long-term reliability under extreme working conditions: Most research focuses on short-term testing at the laboratory scale. However, in battery modules with typical service lifetimes of 8–10 years, thermal barrier materials are required to withstand prolonged thermal cycling, mechanical vibration, and chemical corrosion. Thus, maintaining structural integrity and functional stability under high-temperature shock, continuous flame exposure, and cyclical thermo-mechanical loading is a key challenge for achieving practical applications.
(3) Lightweight design: In high-energy-density battery systems, the mass and thickness of polymer-based thermal insulation materials significantly affect the energy density of the battery pack. Consequently, developing lightweight designs without compromising thermal safety remains a critical research priority.
(4) Scalability and compatibility: Despite the significant progress in polymer-based thermal barrier materials for mitigating TR propagation, several challenges remain for their practical application in battery systems. In particular, scalability and compatibility with real battery modules require further consideration. Most current studies are conducted at the laboratory scale, while large-scale fabrication, cost-effectiveness, and processability remain insufficiently explored. Future efforts should focus on transitioning from complex synthesis to scalable manufacturing. For instance, in polymer-based aerogel systems, thermal insulation performance can be tailored through microstructure design, while scalable fabrication can be achieved by replacing conventional freeze-drying with ambient-pressure drying [155]. In terms of flame retardancy, the development of highly efficient flame-retardant systems with reduced additive loading is essential to maintain processability and lower cost [156]. Moreover, materials should possess sufficient mechanical strength and flexibility to withstand long-term operation, while remaining compatible with conventional manufacturing techniques such as extrusion, coating, or molding. This can be achieved by optimizing polymer network structures, interfacial interactions, and filler dispersion to balance mechanical performance with manufacturability [157].

In conclusion, further research and development are required in the rational design of polymer-based thermal barrier materials for reliable mitigation of TR propagation in LIBs. Multifunctional integration, long-term reliability under extreme working conditions, and lightweight design will be key areas to explore in future investigations. Advancements in these aspects will play a pivotal role in developing highly efficient thermal barrier systems, thereby aligning with the evolving safety and performance demands of next-generation battery technologies.

## Figures and Tables

**Table 1:** Comparison of key properties of representative polymer-based thermal barrier materials.

Polymer Type	Thickness (mm)	Material Form	Thermal Conductivity (W·(m·K)^−1^)	Flame Retardancy	Mechanical Strength (MPa)	TR Propagation Mitigation	Ref.
PI-based	2	Aerogel	0.046	Self-extinguishing	0.970	TR delay 585 s	[51]
Aramid-based	--	Aerogel	0.090	Self-extinguishing	1.410	Reduced heat transfer	[52]
ER-based	3	Porous skeleton	0.830	UL-94 (no rating)	--	--	[53]
PU-based	0.1	Thin film	0.300	pHRR = 108 kW·m^−2^ THR = 16 MJ·m^−2^	--	Stable under 1300 °C flame	[54]
PR-based	--	Aerogel	0.299	LOI = 29.4%	25.60	Thermal buffering	[55]
Silicone-based	3.6	Foam	0.064	LOI = 30.6%	0.026	Backside = 257 °C (1300 °C flame)	[56]
PP-based	2	Solid sheet	0.320	UL-94 V0	100	No propagation	[57]
SBS-based	3	Solid sheet	1.522	LOI = 28.3%	--	Reduce heat accumulation	[58]

## Data Availability

No new data were created or analyzed in this study. Data sharing is not applicable to this article.
